# Mindfulness intervention improves cognitive function in older adults by enhancing the level of miRNA-29c in neuron-derived extracellular vesicles

**DOI:** 10.1038/s41598-021-01318-y

**Published:** 2021-11-08

**Authors:** Shin Hashizume, Masako Nakano, Kenta Kubota, Seiichi Sato, Nobuaki Himuro, Eiji Kobayashi, Akinori Takaoka, Mineko Fujimiya

**Affiliations:** 1 Department of Anatomy, Sapporo Medical University School of Medicine, W17, S1, Chuo-ku, Sapporo, Hokkaido 060-8556 Japan; 2 Department of Physical Therapy, Hokkaido Chitose Rehabilitation College, Chitose, Hokkaido Japan; 3 Division of Signaling in Cancer and Immunology, Institute for Genetic Medicine, Hokkaido University, Sapporo, Hokkaido Japan; 4 Molecular Medical Biochemistry Unit, Biological Chemistry and Engineering Course, Graduate School of Chemical Sciences and Engineering, Hokkaido University, Sapporo, Hokkaido Japan; 5 Department of Public Health, Sapporo Medical University School of Medicine, Sapporo, Hokkaido Japan; 6 Department of Physical Therapy, Faculty of Human Science, Hokkaido Bunkyo University, Eniwa, Hokkaido Japan

**Keywords:** Cognitive ageing, Diseases of the nervous system, Neuroscience

## Abstract

Although mindfulness-based stress reduction (MBSR) improves cognitive function, the mechanism is not clear. In this study, people aged 65 years and older were recruited from elderly communities in Chitose City, Japan, and assigned to a non-MBSR group or a MBSR group. Before and after the intervention, the Japanese version of the Montreal Cognitive Assessment (MoCA-J) was administered, and blood samples were collected. Then, neuron-derived extracellular vesicles (NDEVs) were isolated from blood samples, and microRNAs, as well as the target mRNAs, were evaluated in NDEVs. A linear mixed model analysis showed significant effects of the MBSR x time interaction on the MoCA-J scores, the expression of miRNA(miR)-29c, DNA methyltransferase 3 alpha (DNMT3A), and DNMT3B in NDEVs. These results indicate that MBSR can improve cognitive function by increasing the expression of miR-29c and decreasing the expression of DNMT3A, as well as DNMT3B, in neurons. It was also found that intracerebroventricular injection of miR-29c mimic into 5xFAD mice prevented cognitive decline, as well as neuronal loss in the subiculum area, by down-regulating Dnmt3a  and Dnmt3b  in the hippocampus. The present study suggests that MBSR can prevent neuronal loss and cognitive impairment by increasing the neuronal expression of miR-29c.

## Introduction

Alzheimer’s disease (AD) is a progressive neurodegenerative disorder and is primarily responsible for dementia^1^. The pathological hallmarks of AD are amyloid-β (Aβ) plaques and neurofibrillary tangles in the brain^2^. MicroRNAs (miRNAs) were recently shown to be involved in Aβ production, Aβ-induced neurotoxicity, and tau phosphorylation^3^. MiRNAs are non-coding RNAs 19–25 nucleotides in length that play a role in gene regulation by repressing the translation of target mRNAs^4^.

In the brains of AD patients, the expression of several miRNAs is dysregulated; miRNA (miR)-34a and miR-146a are up-regulated, whereas miRNAs miR-29c and miR-124 are down-regulated^5^. Because the dysregulated expression of miRNAs in the brain may reflect the circulating levels of miRNAs, the blood levels of miR-34a or miR-107 have been used as biomarkers for AD^6,7^. However, circulating miRNAs are a mixture of those released from the brain and other tissues^8^. To detect miRNAs that originate from the brain, neuron-derived extracellular vesicles (NDEVs) in the blood can be isolated using anti-L1 cell adhesion molecule antibody for AD research^9,10^. Extracellular vesicles are released from various kinds of cells and contain miRNA, as well as mRNA, DNA, and proteins^11^. In AD patients, NDEVs in the blood contain higher levels of Aβ and tau, and lower levels of miR-212 compared to a control group^9,10^. However, whether these dysregulated molecules in NDEVs could be normalized by AD treatment is not known.

Treatments for AD are divided into pharmacological and non-pharmacological interventions^12,13^. Among non-pharmacological interventions, evidence has shown that exercise can reduce the risk of developing dementia^14^. In AD model mice, voluntary physical exercise can improve cognitive impairment by suppressing the expression of miR-132 in the hippocampus^15^. We previously reported that an enriched environment (EE) can prevent cognitive impairment in AD and diabetes model animals by increasing the level of miR-146a in the brain and serum^16,17^. An EE is a housing condition that stimulates experience-dependent plasticity due to physical and social surroundings such as toys and mazes^18^. Although an EE contains an element of exercise, an EE is more effective for age-related cognitive impairment, as well as anxiety conditions, than physical exercise^19^. Although an EE cannot be applied directly to humans, exposure to mindfulness meditation, as well as music lessons or physical activities, may partially replicate an EE that has been studied in humans^20^.

Mindfulness-based stress reduction (MBSR) has emerged as a promising non-pharmacological intervention for AD^21^. MBSR is a meditation in which the person pays attention and is aware of his or her ongoing experience in the present moment^22^. There is considerable evidence that a program of MBSR that was designed by Jon Kabat-Zinn in 1979 reduces chronic pain, depression, and anxiety^23–25^. The mechanism of MBSR for psychological improvement involves the regulation of activity in brain regions including the amygdala, anterior cingulate cortex, and dorsolateral prefrontal cortex^26^. The effectiveness of MBSR for mild cognitive impairment (MCI) and AD has been shown in several studies over the last 10 years^27–30^. A randomized study of 120 AD patients in 2016 confirmed the effectiveness of MBSR for maintaining cognitive function^31^.

Although MBSR increases cerebral blood flow and reduces blood inflammatory proteins in older adults with memory loss^27,32^, no previous studies have shown whether MBSR changes levels of miRNA in the brain. Therefore, the purpose of this study was to elucidate the mechanism of the effect of MBSR on cognitive function by focusing on miRNAs in NDEVs.

## Methods

### Participants

This study was approved by the Sapporo Medical University ethics committee (approval number 30-2-17). All methods were carried out according to the principles of the Declaration of Helsinki. Participants aged 65 years and older were recruited from three elderly communities in Chitose City, Japan. After providing an explanation of the study to the participants, written, informed consent was obtained from each one. Participants with severe hypertension, heart disease, psychiatric disorders, and upper and lower limb motor difficulties were excluded.

### Procedure and intervention

**Figure 1:**
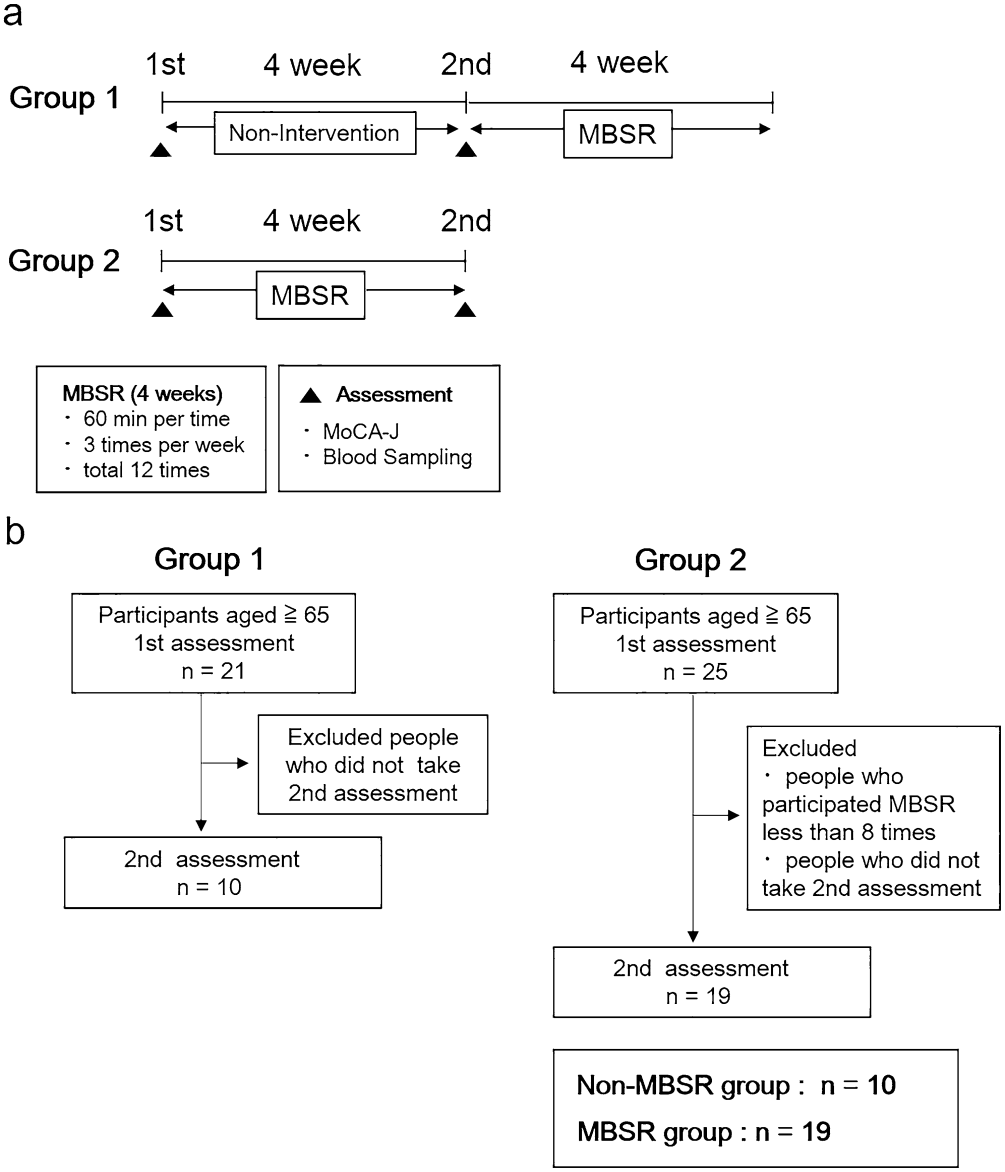
Flow chart of the study design. (**a**) Waiting list controls are assigned to Group 1. In this group, no intervention is performed for 4 weeks, then MBSR is done for 4 weeks. In Group 2, people perform only MBSR for 4 weeks. During the intervention, three MBSR sessions are performed per week for a total of 12 sessions within 4 weeks. Each MBSR session is performed for 60 min. Before and after the intervention, the MoCA-J and blood sampling are conducted. (**b**) A total of 46 older adults aged over 65 years participated in this study (Group 1: n = 21, Group 2: n = 25). The data of participants who performed the MBSR program fewer than eight times or who did not undergo the second assessment were excluded. For the analysis, Group 1 is considered the non-MBSR group (n = 10), and Group 2 is considered the MBSR group (n = 19).

A total of 46 older adults participated in this study. Among the three communities, participants in two communities did the MBSR program after a non-intervention period for four weeks. These people on the waiting list were assigned to Group 1 (n = 21). A waiting list control group is an ethical alternative to having a control group, and they receive the same interventions at a later time^33^. On the other hand, the participants in the other community did only the MBSR program without a non-intervention period and were assigned to Group 2 (n = 25). The participant groups are shown in Fig. 1a. Participants’ age, sex, height, weight, exercise habits (frequency of exercise per week and duration of exercise per session), exercise intensity (frequency of exercise per week x duration of exercise per session), years of education, history of smoking, and number of drinks prior to the intervention were evaluated. During the intervention, three MBSR sessions were performed each week, for a total of 12 sessions within 4 weeks. The CDs of the Japanese translation of the book “Guided Mindfulness Meditation” written by Jon Kabat-Zinn were used^34^. Participants performed a body scan (1st week), yoga meditation (2nd week), sitting meditation (3rd week), and yoga meditation (4th week). A body scan involves paying attention to the body, part by part, noticing different sensations in a gradual sequence from feet to head^35^. On the other hand, yoga and sitting meditation involves expanding the field of awareness to all physical senses and thoughts^35^. Yoga meditation and sitting meditation were performed by adopting designated postures or sitting on a floor, respectively^35^. The sessions were performed for 60 min each time, and yoga postures that are difficult for elderly people were excluded. Before and after the MBSR program in Group 2, cognitive function was assessed using the Japanese version of the Montreal Cognitive Assessment (MoCA-J), and blood samples were collected. The participants in Group1 also underwent the evaluation of MoCA-J and blood sampling before and after the non-intervention period. However we did not perform the assessments after the MBSR in Group 1, because participants in Group 2 did receive only one assessment before the intervention. The data of participants who performed the MBSR program fewer than eight times or who did not undergo the second assessment were excluded. Finally, the data for 10 people in the non-MBSR group and 19 people in the MBSR group were obtained (Fig. 1b).

### Cognitive function test

Cognitive functions were assessed using the MoCA-J, which consists of delayed recall, visuospatial/executive function, attention, abstraction, language, naming, and orientation tasks. The total score and scores for each task were assessed before and after the intervention. Linear mixed models were used for analysis.

### Blood collection

Blood samples were collected from participants using 10-mL vacuum blood collection tubes with heparin (Terumo Corporation, Tokyo, Japan), and plasma was collected 24 h later by centrifugation at 4 °C at 300×g for 10 min and stored at − 80 °C.

### Isolation of total extracellular vesicles

Isolation of total extracellular vesicles was performed according to a previous study^9^. One milliliter of plasma was thawed at 4 °C and then centrifuged at 4 °C at 1500×g for 10 min. Subsequently, 200 µL of plasma were mixed with 400 µL thromboplastin-D (SEKISUI MEDICAL Co., Ltd., Tokyo, Japan) and incubated for 30 min at room temperature. A mixture of protease inhibitor cocktail tablets (Roche, Basel, Switzerland) and phosphatase inhibitor cocktail tablets (Roche) was then added and centrifuged at 4 °C at 3000×g for 30 min. After centrifugation, 600 µL of supernatant were mixed with 151.2 µL ExoQuick (System Biosciences, Palo Alto, CA, USA) and incubated at 4 °C for 30 min. The samples were then centrifuged at 4 °C at 1500×g for 30 min. After centrifugation, all supernatants were removed, and each pellet was stored as total extracellular vesicles at − 80 °C.

### Isolation of NDEVs

Isolation of NDEVs from total extracellular vesicles was performed according to a previous study^9^. Seven hundred microliters of Dulbecco’s phosphate-buffered saline (Thermo Fisher Scientific, Waltham, MA, USA) containing 4 µg Anti-Hu L1 cell adhesion molecule (CD171) Biotin-conjugated Antibody (eBioscience, San Diego, CA, USA) and total extracellular vesicles were mixed and incubated for 1 h at 4 °C. After removing the supernatant, the pellets were suspended in 200 µL 0.05 M Glycine–HCl Buffer (pH 3.0) and centrifuged again at 4 °C at 4000×g for 20 min. The supernatant was mixed with 20 µL 1 M Tris–HCl (pH 8.0), and 100 µL of the mixture of supernatant and Tris–HCl were mixed with 25 µL 3% bovine serum albumin and 365 µL Mammalian Protein Extraction Reagent (Thermo Fisher Scientific). The total products of NDEVs were stored at − 80 °C. We have submitted all relevant data of our experiments to the EV-TRACK knowledgebase^36^ (EV-TRACK ID: EV210240).

### Western blotting

Denatured proteins from NDEV pellets were separated on Mini-PROTEAN AGX Precast Gels (Bio-Rad, Hercules, CA, USA) and transferred to polyvinylidene difluoride (PVDF) membranes. After blocking with 5% skim milk in TBS-T buffer, the membranes were incubated overnight at 4 °C with primary antibodies against CD81 (rabbit polyclonal, 1:1,000, System Biosciences). After washing and incubation with secondary horseradish peroxidase-conjugated goat anti-rabbit IgG (1:10,000, System Biosciences), the blots were developed using Pierce ECL Plus Western Blotting Substrate (Thermo Fisher Scientific, Rockford, IL, USA). Digital images were produced using an Amersham Imager 680 (GE Healthcare Life Sciences, Tokyo, Japan).

### miRNA and mRNA isolation and quantitation

To extract total miRNA and mRNA from total extracellular vesicles and NDEVs, mirVana PARIS RNA and the Native Protein Purification Kit (Thermo Fisher Scientific) were used. The extracted miRNAs were stored at − 80 °C. Fifty nanograms of miRNA were used for the synthesis of cDNA from miRNA using the TaqMan Advanced miRNA cDNA Synthesis kit (Thermo Fisher Scientific). In addition, fifty nanograms of mRNA were used for the synthesis of cDNA from mRNA using the Omniscript RT Kit (QIAGEN, Hilden, Germany). The cDNA was stored at − 20 °C. Real-time PCR of miRNA was performed with the Applied Biosystems 7500 Sequence Detection system (Thermo Fisher Scientific) using TaqMan Fast Advanced Master Mix (Thermo Fisher Scientific). Real-time PCR of mRNA was performed with Applied Biosystems QuantStudio 3 (Thermo Fisher Scientific) using SYBR green (Thermo Fisher Scientific). To determine stable reference genes, the BestKeeper program was used. The value of Ct calculated from the expression of miR-25, miR-93, and miR-425 by BestKeeper was used as a reference gene for the evaluation of miRNA expression in total extracellular vesicles. The value of Ct calculated from the expression of miR-16 and miR-25 by BestKeeper was used as a reference gene for the evaluation of miRNA expression in NDEVs. BestKeeper was also used to calculate the reference Ct from the expression of GAPDH and RNA18S to evaluate the expression of mRNAs in NDEVs. After PCR, ΔCt (Ct target gene – Ct reference gene) was compared before and after the intervention. The primers that targeted miRNA and mRNA are listed in Supplementary Tables 1 and 2. Linear mixed models were used for analysis.

### Luciferase assay

To test the specificity of miR-29 with the 3’ UTR of the identified genes DNMT3A, DNMT3B, STAT3, and BACE1, the 3’ UTR of the gene was cloned downstream of Renilla luciferase gene under the SV40 promoter to perform the luciferase assay. Target sequences of miR-29 in these genes were selected with the online Targetscan program (http://www.targetscan.org/vert_72/). These sequences are shown in Fig. 4a–c, and annealed DNA oligos (Supplementary Table 3) were inserted into the *Xho*I and *Not*I sites of psiCHECK2 vector (Promega, Madison, WI, USA) according to the manual. HEK293T cells (purchased from ATCC, Manassas, VA, USA) were transiently co-transfected with 10 ng of psiCHECK2 including the target site downstream of Renilla luciferase gene and 50 nM of miR-29c (Thermo Fisher Scientific) or negative control (Thermo Fisher Scientific) with Lipofectamine 2000 transfection reagent (Invitrogen). At 24 h after stimulation, luciferase activities were measured with the Dual-Luciferase Reporter Assay system (Promega) according to the manufacturer’s instructions.

### Animals

Animal experiments were performed in accordance with the approved guidelines of the Animal Experiment Committee of Sapporo Medical University (Sapporo, Japan). All experimental protocols were approved by the Animal Experiment Committee of Sapporo Medical University (approval #19-052). The study was carried out in compliance with the ARRIVE guidelines. AD model mice, 5xFAD mice, were bread according to a previous study^17^; they carry five mutations, which are the K670 N/M671L (Swedish), I716V (Florida), and V717I (London) mutations in human amyloid beta precursor protein (APP) and the M146L and L286V mutations in human presenilin 1 (PS1)^37^. In brief, male 5xFAD mice (#034,848, The Jackson Laboratory, Bar Harbor, ME, USA) were crossed with wild-type C57BL/6J females (Sankyo Lab Service Corp., Tokyo, Japan). Then, the needed number of mice for the experiments were obtained. The 5xFAD mice were maintained at a temperature of 21–24 °C and humidity of 50–70%, with a 12-h light/12-h dark cycle. Food and water were available ad libitum.

Every effort was made to eliminate pain and distress in the animals. Isoflurane inhalation and mixed anesthetic agents prepared with 0.3 mg/kg of medetomidine, 4.0 mg/kg of midazolam, and 5.0 mg/kg of butorphanol were used when invasive procedures were needed. The absence of the toe pinch reflex was confirmed before performing the procedures. At the end of the experiments, excess isoflurane inhalation was carried out to euthanize the animals. After confirming cardiopulmonary arrest, all blood was collected from the hearts of the animals.

### Intracerebroventricular injection of miR-29c mimic

At 4 months of age, male 5xFAD mice were implanted with a stainless steel cannula (Eicom, Kyoto, Japan), as described previously^38^. To reach the right ventricle of the brain, the cannula (0.4 mm in diameter) was set 0.4 mm posterior from the bregma, 1 mm right of the midline, and 2.5 mm deep. At 5 months of age, mice were injected with 140 pmol miR-29c mimic (5 µL) or 140 pmol negative control mimic (5 µL) through the cannula with a Hamilton syringe (0.13-mm diameter) at a rate of 1 μL/min. Both the miR-29c mimic and negative control mimic were purchased from Thermo Fisher Scientific, and miRNA complexed with Invivofectamine (Thermo Fisher Scientific) was prepared according to the manufacturer’s instructions. After intracerebroventricular injection four times at 1-week intervals, the Y maze test was conducted.

### Y maze test

The Y-maze (Muromachi Kikai Co., Ltd., Tokyo, Japan) is a three-arm maze with equal angles between all arms, which were 41.5 cm long and 4 cm wide, with walls 10 cm high. The 5xFAD mice were initially placed in one arm and allowed to move freely through the maze. The total numbers of arm entries and alternation behaviors were recorded manually for each mouse over a 7-min period. The alternation score (%) for each mouse was calculated as [(total alternations/total arm entries − 2)] × 100.

### miRNA and mRNA isolation from the hippocampus and quantitation

After the Y maze test, excess isoflurane inhalation was carried out to euthanize the mice. After all blood was collected, brains were removed from the skull and divided into the left and right hemispheres with a scalpel. The left hemisphere was immersed in 4% paraformaldehyde for 24 h and used for immunohistochemical analysis. The right hemisphere was frozen immediately in liquid nitrogen and stored at − 80 °C for PCR analysis. The extraction of miRNA and mRNA and the synthesis of cDNA and PCR were performed as described previously^17^. In brief, the extraction of total miRNA and mRNA from the frozen right hippocampus was performed using the mirVana miRNA isolation kit (Thermo Fisher Scientific). The TaqMan MicroRNA Assay protocol (Thermo Fisher Scientific) or Omniscript RT Kit (QIAGEN) was used for the synthesis of cDNA. Real-time PCR of miRNA was performed with the Applied Biosystems 7500 Sequence Detection system using TaqMan Universal PCR Mastermix II no UNG (Thermo Fisher Scientific). Real-time PCR of mRNA was performed with Applied Biosystems QuantStudio 3 using SYBR green. The relative expressions of miRNA and mRNA were calculated using 2^−ΔΔCt^ with snoRNA 135 or GAPDH as an endogenous control, respectively. The primers that targeted miRNA and mRNA are listed in Supplementary Tables 1 and 4.

### Immunohistochemical analysis

The left hemispheres were placed into 15% sucrose after immersion in 4% paraformaldehyde. The frozen brains of the left hemispheres were cut sagittally into 20-μm-thick sections and used for immunostaining, as described previously^17^. Three sections including the area of the hippocampus (1.4–2.4 mm lateral from the bregma) were chosen and incubated overnight with anti-Aβ antibody or anti-NeuN antibody at 4 °C (listed in Supplementary Table 5). Anti-rabbit IgG FITC or Cy3-labeled antibodies were used as secondary antibodies (listed in Supplementary Table 5). DAPI (Dojindo, Kumamoto, Japan) was used for nuclear staining. Confocal laser scanning microscopy (Nikon, Tokyo, Japan) was used to obtain the images of the subiculum area. The mean of the Aβ-positive area in a 660 × 660 μm^2^ field (one field per section × three sections) and the mean number of NeuN-positive cells in a 200 × 200 μm^2^ field (two fields per section × three sections) in the subiculum area were analyzed.

### Statistical analysis

The analyses of MoCA-J, miRNAs, and mRNAs in NDEVs were performed using a linear mixed model (SPSS Statistics 25) to control potential confounding factors including age, sex, exercise intensity, years of education, and communities. In fitting the model, each variable was scaled, and MBSR and time were entered as fixed effects to test the interaction between MBSR and time. The data for demographics, the delta scores of MoCA-J, the luciferase assay, and the results of the animal experiment were analyzed by the unpaired *t*-test (R version 3.6.1; The R Foundation for Statistical Computing, Vienna. Austria). The demographic data related to sex, smoking and drinking were analyzed by the Chi-squared test (R version 3.6.1). After the analyses, the figures were created using GraphPad Prism 6.0 (GraphPad Software Inc., San Diego, CA, USA).

## Results

### Baseline demographics and characteristics

Table 1 shows the demographics of each group before the MBSR intervention including age, sex, education level, exercise habit, and the MoCA-J score. No significant differences were observed between the non-MBSR and MBSR groups in any items.

**Table 1:** Demographics of the non-MBSR and MBSR groups at baseline.

	Non MBSR group (n = 10)	MBSR group (n = 19)	*p*-value
Age	79.7 ± 4.67	77.26 ± 5.67	*p* = 0.2548
Male/female	1/9	6/13	*p* = 0.197
Height (cm)	154.1 ± 5.45	154.37 ± 7.20	*p* = 0.9187
Body weight (kg)	53.14 ± 6.04	52.76 ± 8.17	*p* = 0.899
BMI (kg/m^2^)	22.35 ± 1.94	22.05 ± 2.31	*p* = 0.7309
Exercise frequency (times per week)	2.9 ± 1.73	2.53 ± 2.27	*p* = 0.5445
Exercise time (min per time)	81.5 ± 63.16	65.79 ± 62.30	*p* = 0.379
Exercise intensity (min per week)	219.5 ± 164.73	184.21 ± 195.71	*p* = 0.3447
Education (years)	10.6 ± 1.51	11.32 ± 1.25	*p* = 0.2349
Smoking, n (%)	0 (0%)	0 (0%)	*p* = 1
Alcohol, n (%)	2 (20%)	7 (37%)	*p* = 0.351
MoCA-J baseline	22.10 ± 3.48	22.89 ± 2.92	*p* = 0.5198

The data of each group for age, sex, height, body weight, body mass index, exercise frequency, exercise duration per session, exercise intensity, education levels, the number of people with smoking or alcohol habits, and the MoCA-J score at baseline are shown. Values are the means ± SD. Non-MBSR group (n = 10), MBSR group (n = 19). No significant differences were observed between the non-MBSR and MBSR groups in any items.

### MBSR improved cognitive function as assessed by MoCA-J

**Figure 2:**
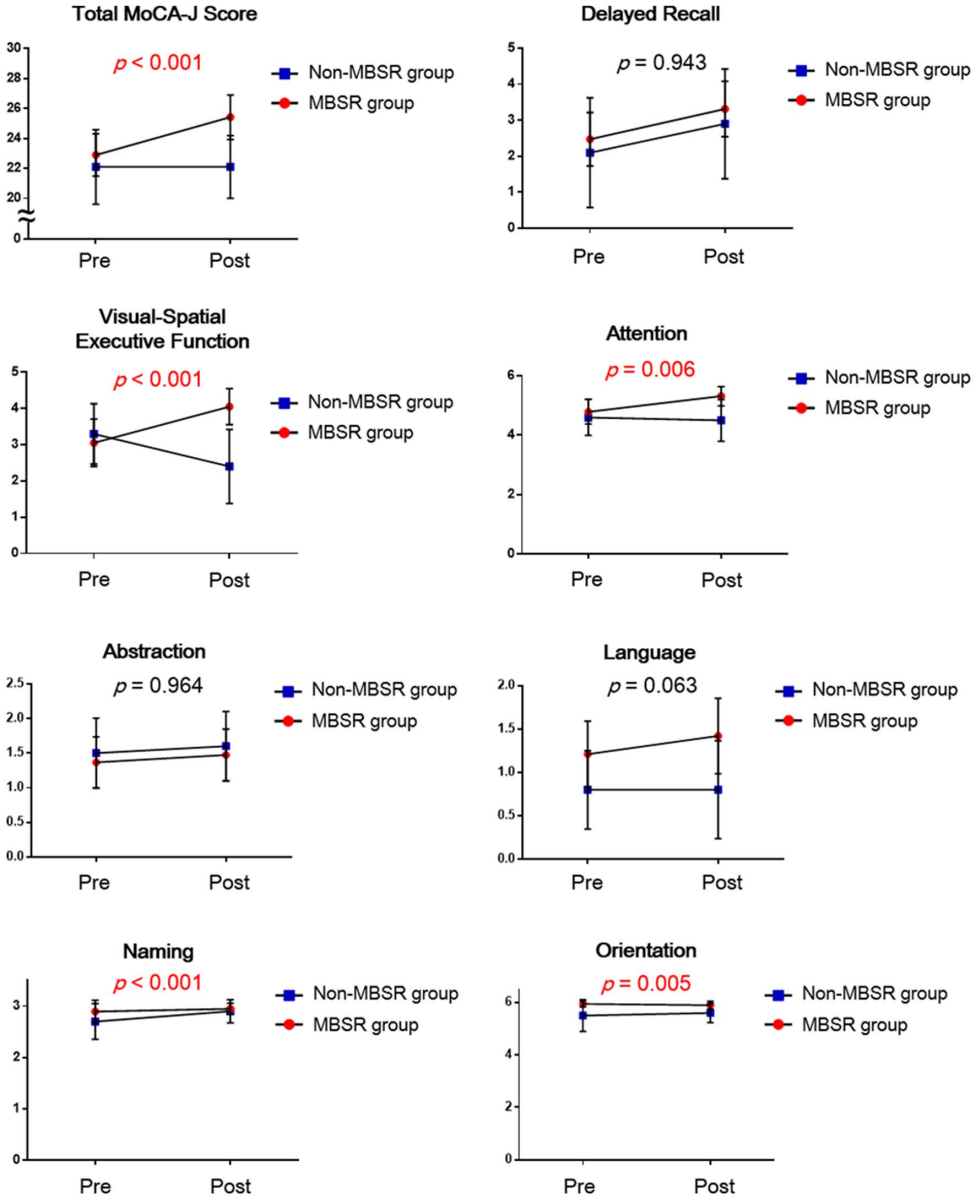
Changes in the scores of the MoCA-J at pre and post intervention in the non-MBSR and MBSR groups. A linear mixed model was used to evaluate the *P* values of the MBSR x time interaction on the total MoCA-J scores and the seven domains of MoCA-J, including delayed recall, visuospatial/executive function, attention, abstraction, language, naming, and orientation. Values are the means ± 95% CI. Non-MBSR group (n = 10), MBSR group (n = 19).

The MoCA-J tests were performed to evaluate cognitive function before and after the intervention in the non-MBSR and MBSR groups. The linear mixed model showed significant effects of the MBSR x time interaction on the total MoCA-J scores, visuospatial/executive function, attention, naming, and orientation (Fig. 2). The analysis of 95% confidence intervals (CIs) of each score showed overlapping 95% CIs in the post scores of the total MoCA-J scores, attention, naming, and orientation. However, there were no overlapping 95% CIs in the post scores of visuospatial/executive function (Fig. 2). The detailed values of the means and 95% CIs are shown in Supplementary Table 6.

The delta scores of MoCA-J (the score of post intervention—the score of pre intervention) were compared between the groups using the *t*-test (Supplementary Fig. 1). There were significant differences in the total MoCA-J scores and the scores of visuospatial/executive function between groups (Supplementary Fig. 1).

### NDEVs were positive for CD81

**Figure 3:**
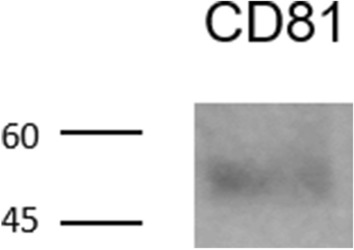
Western blotting for CD81 in NDEVs. NDEVs were isolated from the blood samples, and the presence of the common extracellular vesicle marker of CD81 is confirmed in NDEVs.

After the isolation of NDEVs, the presence of the common extracellular vesicle marker CD81 was confirmed by Western blotting (Fig. 3). The blot image was cropped for clarity of the presentation. The full-length blot was shown in Supplementary Fig. 2.

### MBSR enhanced the expression of miR-29c in NDEVs

Next, the expression of miRNAs in total extracellular vesicles derived from serum was analyzed using the linear mixed model. The expressions of miRNAs related to AD pathogenesis, including miR-9, miR-29c, miR-124, miR-146a, and miR-181a, were analyzed. There were no significant effects of the MBSR x time interaction on any measured miRNA (Supplementary Table 7). Therefore, NDEVs were isolated from serum-derived extracellular vesicles, and the expressions of miRNAs in NDEVs of both groups were analyzed. The linear mixed model showed a significant effect of the MBSR x time interaction on the expression of miR-29c in NDEVs (Table 2). This result suggests that MBSR increases the expression of miR-29c in NDEVs, because the mean ΔCT (post) – ΔCT (pre) of miR-29c was lower in the MBSR group than in the non-MBSR group. The data of 2^-ΔΔCt^ of miRNAs in NDEVs are also shown in Supplementary Table 8.

**Table 2:** Changes in expressions of miRNAs in NDEVs in the non-MBSR and MBSR groups.

miRNA	Non MBSR group (n = 10) ΔCT (post) − ΔCT (pre)	MBSR group (n = 19) ΔCT (post) − ΔCT (pre)	MBSR × Time *p*-value
miR-9	− 0.17 ± 1.05	− 0.70 ± 1.98	*p* = 0.084
miR-29c	− 0.54 ± 0.65	− 1.47 ± 2.30	*p* = 0.003**
miR-124	− 0.28 ± 1.11	− 0.70 ± 1.86	*p* = 0.088
miR-146a	1.06 ± 4.56	0.50 ± 2.83	*p* = 0.686
miR-181a	− 0.12 ± 1.09	− 1.10 ± 2.85	*p* = 0.164

The changes in expressions of miR-9, miR-29c, miR-124, miR-146a, and miR-181a in NDEVs in each group are shown. Values are the means ± SD. A linear mixed model was used to evaluate the *P* values of the MBSR x time interaction. **P < 0.01. Non-MBSR group (n = 10), MBSR group (n = 19).

### MBSR suppressed the expression of DNMT3A, DNMT3B, and BACE1 in NDEVs

Next, target genes that may be affected by an increase in miR-29c were examined. The expressions of DNA methyltransferase 3 alpha (DNMT3A), DNA methyltransferase 3 beta (DNMT3B), signal transducer and activator of transcription 3 (STAT3), and beta-site amyloid precursor protein cleaving enzyme 1 (BACE1) in NDEVs were analyzed. The linear mixed model showed significant effects of the MBSR x time interaction on the expressions of DNMT3A, DNMT3B, and BACE1 in NDEVs (Table 3). These results suggested that MBSR decreases the expressions of DNMT3A, DNMT3B, and BACE1 in NDEVs because the mean ΔCT (post) – ΔCT (pre) values of these genes were higher in the MBSR group than in the non-MBSR group. The data of 2^-ΔΔCt^ of mRNAs in NDEVs are also shown in Supplementary Table 9.

**Table 3:** Changes in expressions of mRNAs in NDEVs in the non-MBSR and MBSR groups.

mRNA	Non MBSR group (n = 10) ΔCT (post) − ΔCT (pre)	MBSR group (n = 19) ΔCT (post) − ΔCT (pre)	MBSR × Time *p*-value
DNMT3A	− 0.09 ± 1.16	1.53 ± 2.30	*p* < 0.001**
DNMT3B	− 0.39 ± 1.31	0.95 ± 1.81	*p* < 0.001**
STAT3	0.18 ± 1.53	0.67 ± 1.67	*p* = 0.28
BACE1	− 0.19 ± 0.78	0.06 ± 0.72	*p* = 0.023*

The changes in expressions of DNMT3A, DNMT3B, STAT3, and BACE1 in NDEVs in each group are shown. Values are the means ± SD. A linear mixed model was used to evaluate the *P* values of the MBSR × time interaction. *P < 0.05, **P < 0.01. Non-MBSR group (n = 10), MBSR group (n = 19).

### Down-regulation of DNMT3A, DNMT3B, and BACE1 by miR-29c in HEK293T cells

**Figure 4:**
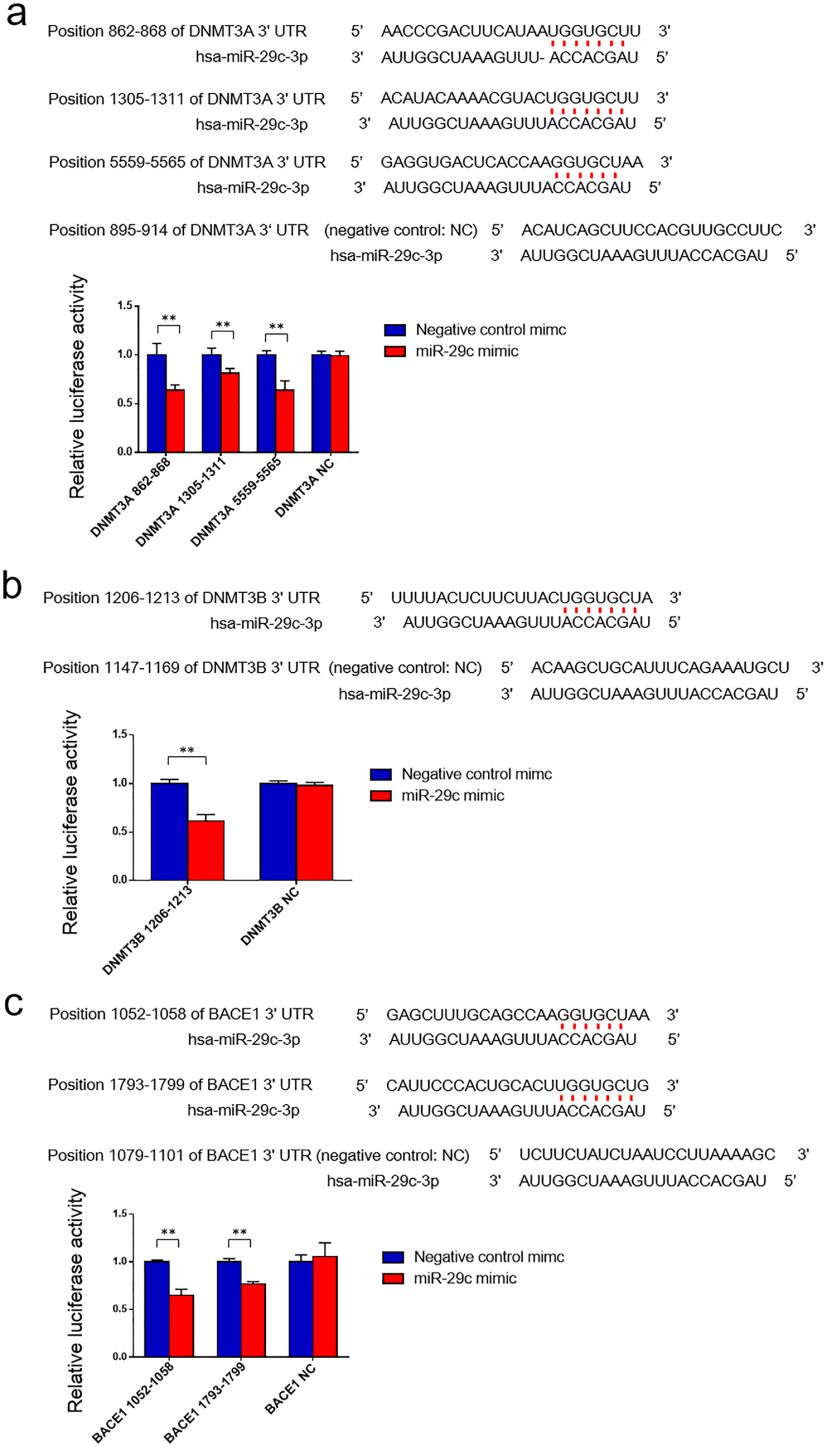
Luciferase assays of miR-29c on DNMT3A, DNMT3B, and BACE1. The binding sites of miR-29c on the 3’UTR regions of DNMT3A, DNMT3B, and BACE1 mRNA are shown in (**a**–**c**), respectively. (**a**–**c**) Luciferase reporter assays show that DNMT3A, DNMT3B, and BACE1 are target genes of miR-29c. Values are the means ± SD. The experiment was repeated four times. **P < 0.01, unpaired *t*-test.

To investigate whether miR-29c decreases the expression of each of DNMT3A, DNMT3B, and BACE1, a luciferase assay was performed using HEK293T cells. Target sequences of miR-29c in these genes were predicted using Targetscan and are shown in Fig. 4a–c. Three positions of DNMT3A, one position of DNMT3B, and two positions of BACE1 were predicted to bind to miR-29c. The assay showed that the miR-29c mimic significantly reduced all DNMT3A-, DNMT3B-, and BACE1-driven luciferase activities in HEK293T cells, though the negative control mimics did not affect their respective activities (Fig. 4a–c). The result of the luciferase assay targeting STAT3 is also shown in Supplementary Fig. 3.

### Intracerebroventricular injection of the miR-29c mimic prevented cognitive impairment in 5xFAD mice

**Figure 5:**
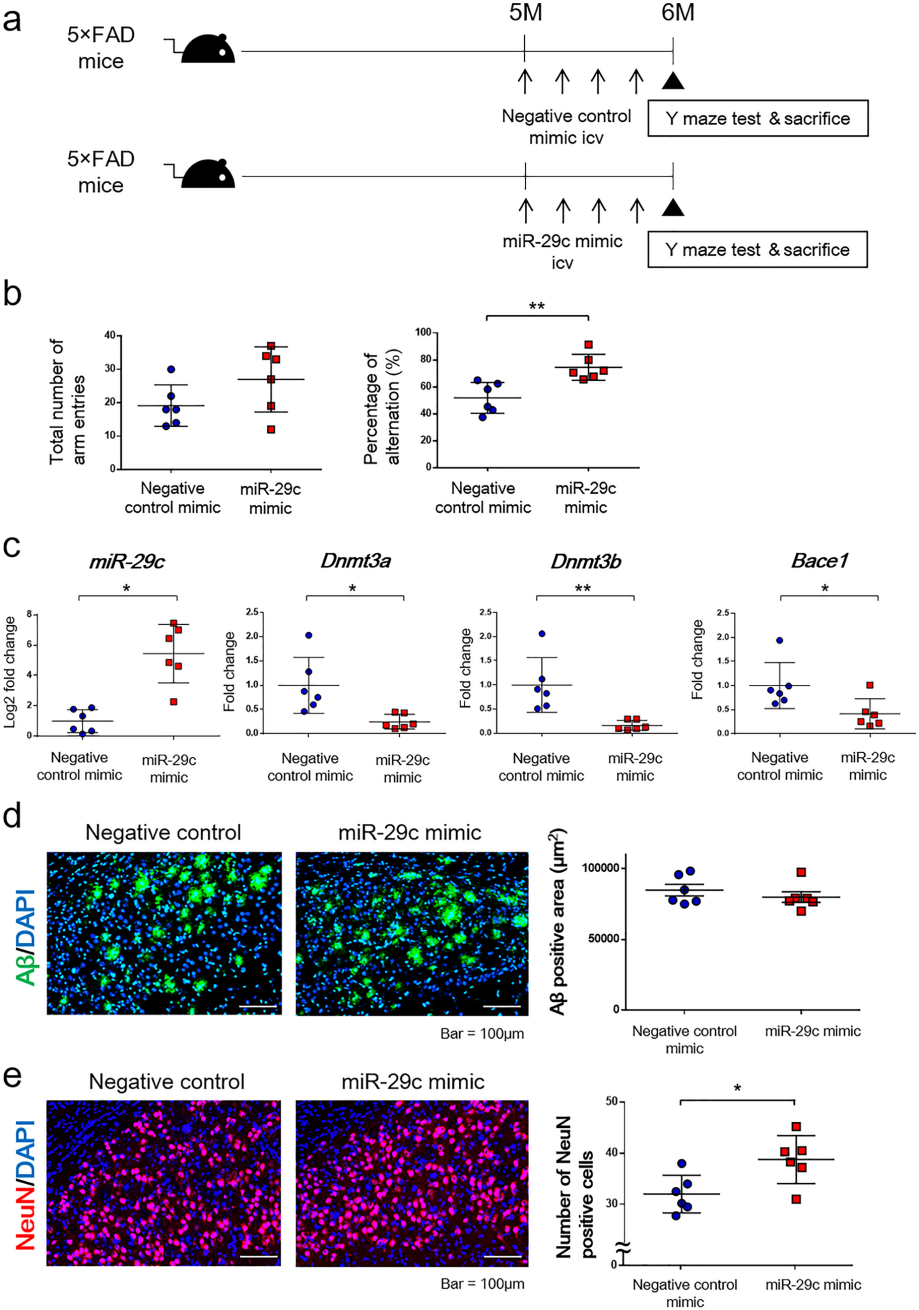
Effect of intracerebroventricular injection of the miR-29c mimic into 5xFAD mice. (**a**) Experimental protocol. Negative control miRNA (140 pmol) or miR-29c mimic (140 pmol) is injected intracerebroventricularly into 5-month-old 5xFAD mice four times at 1-week intervals. At 1 week after the last injection, Y maze tests are conducted, and mice are sacrificed at 6 months of age. (**b**) The results of Y maze tests. Total numbers of arm entries and the percentages of alternations are shown. Values are the means ± SEM. *P < 0.05, unpaired *t*-test. Negative control mimic group (n = 6), miR-29c mimic group (n = 6). (**c**) The expressions of miR-29c, Dnmt3a, Dnmt3b, and Bace1. Values are the means ± SEM. *P < 0.05, unpaired *t*-test. Negative control mimic group (n = 6), miR-29c mimic group (n = 6). (**d**) The Aβ-positive area. Values are the means ± SEM. Negative control mimic group (n = 6), miR-29c mimic group (n = 6). (**e**) The number of NeuN-positive cells. Values are the means ± SEM. *P < 0.05, unpaired *t*-test. Negative control mimic group (n = 6), miR-29c mimic group (n = 6).

To investigate the type of molecular and pathological changes that occur due to the increase in miR-29c in the central nervous system (CNS), the miR-29c mimic was injected intracerebroventricularly into AD model mice. The 5xFAD mice that have three mutations in amyloid precursor protein (APP) and two mutations in presenilin-1 (PSEN1) were used^37^. The 5xFAD mice show no cognitive impairment at 5 months of age, but they show cognitive decline beginning at 6 months of age (Supplementary Fig. 4a, b). To investigate whether injection of the miR-29c mimic prevents a decline in cognitive function, negative control miRNA or miR-29c mimic (140 pmol/animal) was injected into 5-month-old 5xFAD mice four times at 1-week intervals. One week after the last injection, Y maze tests were conducted, and mice were sacrificed at 6 months of age for further analysis (Fig. 5a).

In the Y maze tests, the total number of arm entries was not different between the two groups. However, the percentage of alternations, which represents cognitive function, was significantly higher in the miR-29c mimic group than in the negative control group (Fig. 5b).

### The increase in miR-29c in the CNS did not affect the level of Aβ, but prevented the decrease in neuronal number in the hippocampus by down-regulating the levels of Dnmt3a and Dnmt3b

The mechanisms by which the miR-29c mimic prevents cognitive impairment in 5xFAD mice were investigated. The hippocampal expression of miR-29c was significantly increased in the miR-29c mimic-injected group compared to the negative control group. On the other hand, the hippocampal expressions of Dnmt3a, Dnmt3b, and Bace1 were significantly decreased in the miR-29c mimic-injected group compared to the negative control group (Fig. 5c).

Because BACE1 is the enzyme that cleaves APP at the β-site and produces Aβ^39^, the expression of Aβ in the subiculum area was evaluated. However, there were no significant differences in the positive area of Aβ between the groups (Fig. 5d).

Because decreased expression of DNMT3A inhibits neuronal apoptosis^40^, the number of NeuN-positive cells in the subiculum area was evaluated. The number of NeuN-positive cells was significantly higher in the miR-29c mimic-injected group than in the negative control group (Fig. 5e).

## Discussion

To the best of our knowledge, this is the first report to show the mechanism behind the effect of MBSR on cognitive function by focusing on miRNA. To evaluate how MBSR changes the expression of miRNAs in the brain, NDEVs isolated from blood were examined. It was found that MBSR not only improved cognitive function in older adults, but also increased the level of miR-29c in NDEVs. In addition, MBSR decreased the expressions of DNMT3A, DNMT3B, and BACE1, which are target genes of miR-29c, in NDEVs. Furthermore, intracerebroventricular injection of miR-29c improved cognitive function and inhibited neuronal apoptosis in 5xFAD mice by down-regulating the expression of Dnmt3a and Dnmt3b in the hippocampus. Therefore, MBSR may have an effect on preserving neuronal number by increasing the expression of miR-29c in the CNS.

MBSR improves cognitive function in MCI and AD patients^21,27,29–31^. Although MBSR is typically an 8-week program, MBSR was performed for 4 weeks because this shortened version of MBSR is fully effective for psychological problems including stress^41,42^. Jon Kabat-Zinn recommends a program of 8 weeks that consists of a body scan (1–2 weeks), the combination of a body scan and yoga meditation (3–4 weeks), the combination of yoga and sitting meditation (5–6 weeks), and a free combination of a body scan, yoga, or sitting meditation (7–8 weeks)^35^. In this study, this 8-week program was shortened to a 4-week program that consists of a body scan (1st week), yoga meditation (2nd week), sitting meditation (3rd week), and yoga meditation (4th week).

To evaluate cognitive function, the Japanese version of the MoCA that was originally written in English by Nasreddine et al. in 2005 was used^43,44^. The MoCA was developed as a brief screening test for MCI to address the limitations of the Mini-Mental State Examination^44^. In the present study, the MoCA-J score of all participants at baseline was 22.6 ± 3.1 (mean ± SD). Rossetti et al. reported that the MoCA score of people of different races aged 70–80 years was 21.32 ± 4.78 (mean ± SD)^45^; the MoCA-J score in Japanese people aged 85–87 years was 22.9 ± 3.5 (mean ± SD)^46^. Considering that the age of the participants in this study was 78.1 ± 5.4 years (mean ± SD), their cognitive function did not seem to be significantly different from that reported in previous studies.

There was a significant effect of the MBSR x time interaction on the total MoCA-J scores. In the MBSR group, the delta score of the total MoCA-J (the score of post intervention—the score of pre intervention) was 2.53. In a previous study^47^, the minimum clinically important difference of MoCA was found to be 2. Therefore, MBSR appears to increase cognitive function effectively.

In the analysis of the sub-categories of MoCA-J, there was a significant effect of the MBSR x time interaction on visuospatial/executive function. Since there were no overlapping 95% CIs in the post scores of visuospatial/executive function, MBSR appears to increase the visuospatial/executive function effectively. A previous study has already shown that brief meditation training improves visuospatial processing and executive functioning^48^. Therefore, MBSR might have improved visuospatial/executive function more than the other sub-classified functions of MoCA-J. In addition, there were significant effects of the MBSR x time interaction on attention, naming, and orientation. However, this does not suggest that MBSR actually changes these functions, because there were overlapping 95% CIs in the post scores of these functions.

To investigate the effects of MBSR on neuronal miRNA expressions, NDEVs were isolated from the blood samples. In the present study, the presence of the common extracellular vesicle marker of CD81 in NDEVs was confirmed by Western blotting. The results were consistent with previous studies^9,49^. Although the sizes of NDEVs were not measured, the previous study identified NDEVs as cup-shaped structures, 30–100 nm in size, by transmission electron microscopy^49^.

In previous studies, both cerebral expression of miR-29c and its level in blood were shown to be decreased in AD patients^50,51^. Because the expression of miR-29c in NDEVs was up-regulated by MBSR in the present study, the neuronal expression of miR-29c in the CNS is likely to be increased.

Because miR-29c suppresses the target genes of DNMT3A, DNMT3B, STAT3, and BACE1^40,50,51^, the expressions of these genes in NDEVs were analyzed. The MBSR intervention was found to decrease the expressions of DNMT3A, DNMT3B, and BACE1. To investigate whether miR-29c functionally down-regulates the expressions of DNMT3A, DNMT3B, and BACE1, luciferase assays were performed in human HEK293T cells. It was demonstrated that the miR-29c mimic decreased the expressions of DNMT3A, DNMT3B, and BACE1. Therefore, neuronal expression of miR-29c appears to down-regulate these mRNA expressions.

DNMT3A and DNMT3B are the main DNA methyltransferases that initiate DNA methylation in neurons^52^. DNA methylation is a crucial epigenetic marker for the regulation of gene transcription^53^. The level of miR-29c is decreased in a rat ischemic brain damage model, and treatment with miR-29c inhibits neuronal cell death by suppressing DNMT3A^40^. Because the expression of miR-29c was increased and that of DNMT3A was decreased in NDEVs in the present study, neuronal death is likely to be inhibited by MBSR. Additionally, DNMT3A and DNMT3B are increased in 5xFAD model mice^54^, and higher cytosine methylation is related to cognitive impairment^54^. Because the MBSR intervention decreased the expressions of DNMT3A and DNMT3B in NDEVs, these molecular changes may be involved in the up-regulation of gene transcription that promotes cognitive function. BACE1 is an enzyme that cleaves APP and produces Aβ^39^. The level of Aβ in NDEVs was measured, but the levels of Aβ were under the detection limit (data not shown). Thus, whether MBSR decreases the level of Aβ in NDEVs by down-regulating BACE1 could not be determined.

In the present study, the expressions of miRNAs including miR-9, miR-124, miR-146a, and miR-181a in NDEVs were not changed by MBSR. MiR-9 and miR-124 play a role in repression of BACE1, and both miRNAs are down-regulated in the brain of AD patients^5^. Because no significant changes were observed in miR-9 and miR-124, these miRNAs may not be involved in the decreased expression of BACE1. MiR-146a has an anti-inflammatory effect by suppressing the expression of NF-κB; however, miR-146a is increased in the brains of AD patients^55^. We previously reported that intracerebroventricular injection of mesenchymal stem cells improves cognitive impairment in AD model mice by transferring exosomal miR-146a into the CNS^38^. In the present study, there was no significant change in the expression of miR-146a in NDEVs, suggesting that miR-146a may not be involved in the cognitive improvement with MBSR. MiR-181a enhances hippocampus-dependent memory by targeting protein kinase AMP-activated catalytic subunit alpha 1 (PRKAA1)^56^. Because no difference was found in the expression of miR-181a, MBSR may not affect the expression of PRKAA1.

To investigate why neuronal up-regulation of miR-29c by MBSR is related to cognitive improvement, an miR-29c mimic was injected intracerebroventricularly into 5xFAD mice. The Y maze test was then conducted to assess short-term memory^57^. It was found that the up-regulation of miR-29c prevented cognitive impairment in 5xFAD mice, possibly by inhibiting the expressions of DNMT3A, DNMT3B, and BACE1 in the hippocampus. Though the luciferase assays were not performed using murine cells, luciferase assays using mouse 3T3-L1 cells have shown that miR-29c suppressed the expression of DNMT3A^58^. Similarly, luciferase assays using mouse neuronal cells have also shown that miR-29c suppressed the expression of BACE1^59^. However, the decrease in BACE1 in mice may not have affected the production of Aβ, because the Aβ-positive area in the subiculum was not changed by up-regulation of miR-29c. Because 5xFAD is a model that has five mutations and rapidly develops severe amyloid pathology, decreased expression of BACE1 may not be sufficient to reduce Aβ.

On the other hand, the number of NeuN-positive cells was significantly higher in the miR-29c mimic-injected group. Because down-regulation of DNMT3A inhibits neuronal apoptosis^40^, these molecular changes may have a positive effect on preservation of neuronal number. We previously reported that preservation of neuronal number is associated with normal cognitive function in human subjects with severe accumulation of Aβ and tau in the brain^60^. Therefore, inhibited neuronal death in the miR-29c mimic-injected group may have contributed to the prevention of cognitive decline, despite no decline in Aβ. Thus, MBSR may have an effect in inhibiting neuronal apoptosis by up-regulating miR-29c in neurons.

Although a new mechanism regarding the effectiveness of MBSR on cognitive function was identified, how epigenetic changes were induced by the decreases in DNMT3A and DNMT3B was not determined. The increase in DNA methylation of the brain-derived neurotrophic factor (BDNF) promoter may be responsible for the reduction in BDNF mRNA or protein in AD patients^61,62^. In addition, enhanced DNA methylation accompanied by an increase in DNMT3 is related to a decrease in miR-29c in AD patients^62^. Therefore, the up-regulation of miR-29c induced by MBSR may have enhanced the transcription of BDNF by down-regulating DNMT3A and DNMT3B.

This is the first study to show that MBSR improves cognitive function by up-regulating the expression of miR-29c in NDEVs. However, there are several limitations to take into account. First, the number of participants in the present study was small. Second, this was not a randomized, controlled study. Finally, the duration of MBSR was shortened from 8 to 4 weeks. For these reasons, the possibility of a transient short-term change in cognitive scores cannot be ruled out. However, mindfulness is known to improve inflammatory biomarker levels in older adults with MCI up to 9-month follow-up^32^. In the present study, though one has to be careful drawing conclusions, significant changes in the expressions of miR-29c, DNMT3A, and DNMT3B in NDEVs were found. In the future, we would like to confirm these results by increasing the number of participants.

Among non-pharmacological interventions, exercise is a well-known method to reduce the risk of AD^14^. Exercise increases the level of miR-21 in serum^63^ and enhances epigenetic changes including DNA methylation in the CNS^64^. Although MBSR involves slight body movement, the average energy expenditures during yoga and meditation are lower than during walking^65^. Thus, the physical element of MBSR may not have contributed to the change in miR-29c in NDEVs.

In summary, MBSR improved cognitive function in older adults possibly by increasing neuronal expression of miR-29c. Mesenchymal stem cell-derived exosomes containing miR-29b and miR-146a are effective in AD models by transferring the exosomes into the CNS^38,66^. However, it was found that MBSR can increase the neuronal expression of miR-29c without the injection of stem cells or exosomes. These findings suggest that mindfulness can prevent the onset of dementia and regulate miRNAs in the body, even without pharmacological interventions. Further evidence is needed to establish MBSR as a method for preventing or treating cognitive impairment.

## Supplementary Information

Supplementary Information.
