# Supplementary material for: Mindfulness intervention improves cognitive function in older adults by enhancing the level of miRNA-29c in neuron-derived extracellular vesicles
Source: Sci Rep. 2021 Nov 8;11:21848. doi: 10.1038/s41598-021-01318-y (PMC8575875; doi:10.1038/s41598-021-01318-y)
Supplement: Supplementary file 1 — Supplementary Information. [file 41598_2021_1318_MOESM1_ESM.pdf]

Mindfulness intervention improves cognitive function in older adults by enhancing the level of miRNA-29c in neuron-derived extracellular vesicles

Shin Hashizume[1], Masako Nakano[1]*, Kenta Kubota[1,2], Seiichi Sato[3,4], Nobuaki Himuro[5], Eiji Kobayashi[1,6], Akinori Takaoka[3,4], Mineko Fujimiya[1]

[1] Department of Anatomy, Sapporo Medical University School of Medicine, Sapporo, Hokkaido, Japan
[2] Department of Physical Therapy, Hokkaido Chitose Rehabilitation College, Chitose, Hokkaido, Japan
[3] Division of Signaling in Cancer and Immunology, Institute for Genetic Medicine, Hokkaido University, Sapporo, Hokkaido, Japan
[4] Molecular Medical Biochemistry Unit, Biological Chemistry and Engineering Course, Graduate School of Chemical Sciences and Engineering, Hokkaido University, Sapporo, Hokkaido, Japan
[5] Department of Public Health, Sapporo Medical University, School of Medicine, Sapporo, Hokkaido, Japan
[6] Department of Physical Therapy, Faculty of Human Science, Hokkaido Bunkyo University, Eniwa, Hokkaido, Japan

*Corresponding author

Supplementary information

1. Supplementary Tables
2. Supplementary Figures

Supplementary Table 1

PCR primers for miRNA analysis

Gene	Sequence
miRNA-9-5p	5'-UCUUUGGUUAUCUAGCUGUAUGA-3'
miRNA-29c-3p	5'-UAGCACCAUUUGAAAUCGGUUA-3'
miRNA-124-3p	5'-UAAGGCACGCGGUGAAUGCC-3'
miRNA-125b-5p	5'-UCCCUGAGACCCUAACUUGUGA-3'
miRNA-146a-5p	5'-UGAGAACUGAAUUCCAUGGGUU-3'
miRNA-181a-5p	5'-AACAUUCAACGCUGUCGGUGAGU-3'
miRNA-25-3p	5'-CAUUGCACUUGUCUCGGUCUGA-3'
miRNA-93-5p	5'-CAAAGUGCUGUUCGUGCAGGUAG-3'
miRNA-425-5p	5'-AAUGACACGAUCACUCCCGUUGA-3'
miRNA-16-5p	5'-UAGCAGCACGUAAAUAUUGGCG-3'
snoRNA135	5'-CUAAAAUAGCUGGAAUUACCGGCAGAUUGGUAGUGGUGA GCCUAUGGUUUUCUGAAG-3'

Supplementary Table 1. Target sequences of miRNAs and snoRNA 135.

Supplementary Table 2

mRNA PCR primers for human

Gene	Direction	Sequence
DNMT3A	forward	5’-TATTGATGAGCGCACAAGAGAGC-3’
	reverse	5’-GGGTGTTCCAGGGTAACATTGAG-3’
DNMT3B	forward	5’-GACTTGGTGATTGGCGGAA-3’
	reverse	5’-GGCCCTGTGAGCAGCAGA-3’
STAT3	forward	5’-CTTTGAGACCGAGGTGTATCACC-3’
	reverse	5’-GGTCAGCATGTTGTACCACAGG-3’
BACE1	forward	5’-GCAGGGCTACTACGTGGAGA-3’
	reverse	5’-GTATCCACCAGGATGTTGAGC-3’
GAPDH	forward	5’-GTCTCCTCTGACTTCAACAGCG-3’
	reverse	5’-ACCACCCTGTTGCTGTAGCCAA-3’
RNA18s	forward	5’-GTAACCCGTTGAACCCCATT-3’
	reverse	5’-CCATCCAATCGGTAGTAGCG-3’

Supplementary Table 2. Sequence of primers for human mRNAs.

Supplementary Table 3

DNA oligos for luciferase assay

Gene	Direction	Sequence
Position 862-868 of DNMT3A 3' UTR	forward	5’ TCGAGGTTAACTACTGTACAACCCGACTTCATAATGGTGCTTTCGC 3’
	reverse	5’ GGCCGCGAAAGCACCATTATGAAGTCGGGTTGTACAGTAGTTAACC 3’
Position 1305-1311 of DNMT3A 3' UTR	forward	5’ TCGAGCCCCACCTGGAGCAAATAAAAAAACATACAAAACGTACTGGTGCTTTGC 3’
	reverse	5’ GGCCGCAAAGCACCAGTACGTTTTGTATGTTTTTTTATTTGCTCCAGGTGGGGC 3’
Position 5559-5565 of DNMT3A 3' UTR	forward	5’ TCGAGCCTTAGAATACGCAGAGGGAGGAGGTGACTCACCAAGGTGCTAAAAGC 3’
	reverse	5’ GGCCGCTTTTAGCACCTTGGTGAGTCACCTCCTCCCTCTGCGTATTCTAAGGC 3’
Position 895-914 of DNMT3A 3‘ UTR (negative control)	forward	5’ TCGAGACATCAGCTTCCACGTTGCCTTCTGCGCAAAGGGTTTCAGC 3’
	reverse	5’ GGCCGCTGAAACCCTTTGCGCAGAAGGCAACGTGGAAGCTGATGTC 3’
Position 1206-1213 of DNMT3B 3' UTR	forward	5’ TCGAGTCATAATGGTTTTTAACACCTTTTACTCTTCTTACTGGTGCTATTTGC 3’
	reverse	5’ GGCCGCAAATAGCACCAGTAAGAAGAGTAAAAGGTGTTAAAAACCATTATGAC 3’
Position 1147-1169 of DNMT3B 3' UTR (negative control)	forward	5’ TCGAGACAAGCTGCATTTCAGAAATGCTGTCATAATGGTTTTTAACACCTTGC 3’
	reverse	5’ GGCCGCAAGGTGTTAAAAACCATTATGACAGCATTTCTGAAATGCAGCTTGTC 3’
Position 1052-1058 of BACE1 3' UTR	forward	5’ TCGAGCAAATCTTCCTCTGGAGCTTTGCAGCCAAGGTGCTAAAAGC 3’
	reverse	5’ GGCCGCTTTTAGCACCTTGGCTGCAAAGCTCCAGAGGAAGATTTGC 3’
Position 1793-1799 of BACE1 3' UTR	forward	5’ TCGAGGTTTTTTATCTGGGTTCTCTTCATTCCCACTGCACTTGGTGCTGC 3’
	reverse	5’ GGCCGCAGCACCAAGTGCAGTGGGAATGAAGAGAACCCAGATAAAAAACC 3’
Position 1079-1101 of BACE1 3' UTR (negative control)	forward	5’ TCGAGTCTTCTATCTAATCCTTAAAAGCATAATGTTGAACATTCGC 3’
	reverse	5’ GGCCGCGAATGTTCAACATTATGCTTTTAAGGATTAGATAGAAGAC 3’
Position 1011-1017 of STAT3 3' UTR	forward	5’ TCGAGAAAAAAAAAAAAAAAAAAAAAGAAACTTCAGTTAACAGCCTCCTTGGTGCTTTGC 3’
	reverse	5’ GGCCGCAAAGCACCAAGGAGGCTGTTAACTGAAGTTTCTTTTTTTTTTTTTTTTTTTTC 3’
Position 895-917 of STAT3 3' UTR (negative control)	forward	5’ TCGAGAGGTTGCAGTGAGCCAAAATTGCACCACTGCACACTGCACTCCATCCTGGGCGC 3’
	reverse	5’ GGCCGCGCCCAGGATGGAGTGCAGTGTGCAGTGGTGCAATTTTGGCTCACTGCAACCTC 3’

Supplementary Table 3. Sequence of DNA oligos for luciferase assay.

Supplementary Table 4

mRNA PCR primers for mouse

Gene	Direction	Sequence
Dnmt3a	forward	5’-GCCGAATTGTGTCTTGGTGGATGACA-3’
	reverse	5’-CCTGGTGGAATGCACTGCAGAAGGA-3’
Dnmt3b	forward	5’-TTCAGTGACCAGTCCTCAGACACGAA-3’
	reverse	5’-TCAGAAGGCTGGAGACCTCCCTCTT-3’
Stat3	forward	5’-CTTGTCTACCTCTACCCCGACAT-3’
	reverse	5’-GATCCATGTCAAACGTGAGCG-3’
Bace1	forward	5’-CCGGCGGGAGTGGTATTATG-3’
	reverse	5’-GCAAACGAAGGTTGGTGGT-3’
Gapdh	forward	5’-ACGACCCCTTCATTGACC-3’
	reverse	5’-CCAGTGAGCTTCCCGTTCAGC-3’

Supplementary Table 4. Sequence of primers for mouse mRNAs.

Supplementary Table 5

Immunofluorescence

Primary Antibody	Source	Dilution	Manufacture
Aβ	rabbit monoclonal	1:500	Cell Signaling
NeuN	rabbit polyclonal	1:500	Millipore

Secondary Antibody	Conjugate	Dilution	Manufacture
Rabbit IgG	FITC	1:500	Millipore
Rabbit IgG	Cy3	1:500	Jackson ImmunoResearch

Supplementary Table 5. Primary and secondary antibodies used for immunofluorescence.

Supplementary Table 6

MoCA-J	Time-points	Non-MBSR group mean (SD, 95%CI)	MBSR group mean (SD, 95%CI)	MBSR x Time *p*-value
Total score	pre	22.10 (3.48, 19.61 to 24.59)	22.89 (2.92, 21.49 to 24.30)	$p < 0.001$
	post	22.10 (2.92, 20.01 to 24.19)	25.42 (3.08, 23.94 to 26.91)	
Delayed recall	pre	2.10 (2.13, 0.58 to 3.62)	2.47 (1.54, 1.73 to 3.22)	$p = 0.943$
	post	2.90 (2.13, 1.38 to 4.42)	3.32 (1.60, 2.54 to 4.09)	
Visuospatial/ executive function	pre	3.30 (1.16, 2.47 to 4.13)	3.05 (1.35, 2.40 to 3.70)	$p < 0.001$
	post	2.40 (1.43, 1.38 to 3.42)	4.05 (1.03, 3.56 to 4.55)	
Attention	pre	4.60 (0.84, 4.00 to 5.20)	4.79 (0.85, 4.38 to 5.20)	$p = 0.006$
	post	4.50 (0.97, 3.80 to 5.20)	5.32 (0.67, 4.99 to 5.64)	
Abstraction	pre	1.50 (0.71, 0.99 to 2.01)	1.37 (0.76, 1.00 to 1.74)	$p = 0.964$
	post	1.60 (0.70, 1.10 to 2.10)	1.47 (0.77, 1.10 to 1.85)	
Language	pre	0.80 (0.63, 0.35 to 1.25)	1.21 (0.79, 0.83 to 1.59)	$p = 0.063$
	post	0.80 (0.79, 0.24 to 1.36)	1.42 (0.90, 0.99 to 1.86)	
Naming	pre	2.70 (0.48, 2.35 to 3.05)	2.89 (0.46, 2.67 to 3.12)	$p < 0.001$
	post	2.90 (0.32, 2.67 to 3.13)	2.95 (0.23, 2.84 to 3.06)	
Orientation	pre	5.50 (0.85, 4.89 to 6.11)	5.95 (0.23, 5.84 to 6.06)	$p = 0.005$
	post	5.60 (0.52, 5.23 to 5.97)	5.89 (0.32, 5.74 to 6.05)	

Supplementary Table 6. The results of MoCA-J. non-MBSR group (n = 10), MBSR group (n = 19). Values are the means, SD, and 95%CI. Linear mixed model was used to evaluate the *P* values of MBSR x time interaction.

Supplementary Table 7

miRNA (total)	Non MBSR group (n=10) ΔCT (post) – ΔCT (pre)	MBSR group (n=19) ΔCT (post) – ΔCT (pre)	MBSR x Time *p*-value
**miR-9**	-0.02 ± 0.64	-0.09 ± 0.83	p = 0.826
**miR-29c**	0.04 ± 0.59	-0.07 ± 0.70	p = 0.673
**miR-124**	0.04 ± 0.47	-0.03 ± 0.81	p = 0.537
**miR-146a**	0.06 ± 0.54	-0.10 ± 0.61	p = 0.228
**miR-181a**	0.37 ± 0.68	-0.05 ± 0.41	p = 0.055

Supplementary Table 7. The changes in the expression of miR-9, miR-29c, miR-124, miR-146a, and miR-181a in total extracellular vesicles in each group are shown. Values are the means ± SD. Linear mixed model was used to evaluate the *P* values of MBSR x time interaction. Non-MBSR group (n = 10), MBSR group (n = 19).

Supplementary Table 8

miRNA	Non MBSR group (n=10) $2^{(-\Delta\Delta Ct)}$	MBSR group (n=19) $2^{(-\Delta\Delta Ct)}$	Non-MBSR vs MBSR group *p*-value
	Median (Q1, Q3)	Median (Q1, Q3)	
**miR-9**	1.1668 (0.6033, 1.7387)	1.5864 (0.8216, 2.4860)	$p = 0.4557$
**miR-29c**	1.5366 (0.9733, 1.7073)	1.8919 (1.3992, 4.1883)	$p = 0.211$
**miR-124**	1.2998 (0.6714, 1.9382)	1.4410 (0.7629, 2.9638)	$p = 0.5421$
**miR-146a**	0.2265 (0.0815, 3.3618)	0.8831 (0.4094, 1.1407)	$p = 0.4557$
**miR-181a**	0.9428 (0.6674, 1.5892)	1.7704 (0.9793, 4.0459)	$p = 0.179$

Supplementary Table 8. The expression $2^{(-\Delta\Delta Ct)}$ of miR-9, miR-29c, miR-124, miR-146a, and miR-181a in NDEVs in each group are shown. Values are the median, Q1 and Q3. Unpaired *t*-test. Non-MBSR group (n = 10), MBSR group (n = 19).

Supplementary Table 9

mRNA	Non MBSR group (n=10) $2^{(-\Delta\Delta Ct)}$	MBSR group (n=19) $2^{(-\Delta\Delta Ct)}$	Non-MBSR vs MBSR group *p*-value
	Median (Q1, Q3)	Median (Q1, Q3)	
**DNMT3A**	1.0467 (0.7172, 1.0855)	0.5223 (0.2096, 0.8314)	*p* = 0.03515 *
**DNMT3B**	1.2052 (0.5609, 1.8554)	0.6864 (0.3454, 1.3093)	*p* = 0.1946
**STAT3**	0.6914 (0.4269, 1.3698)	0.6505 (0.3530, 1.3854)	*p* = 0.8747
**BACE1**	1.1068 (0.7233, 1.6875)	0.9914 (0.8442, 1.1895)	*p* = 0.6683

Supplementary Table 9. The expression $2^{(-\Delta\Delta Ct)}$ of DNMT3A, DNMT3B, STAT3 and BACE1 in NDEVs in each group are shown. Values are the median, Q1 and Q3. *$P < 0.05$, unpaired *t*-test. Non-MBSR group (n = 10), MBSR group (n = 19).

Supplementary Figure 1

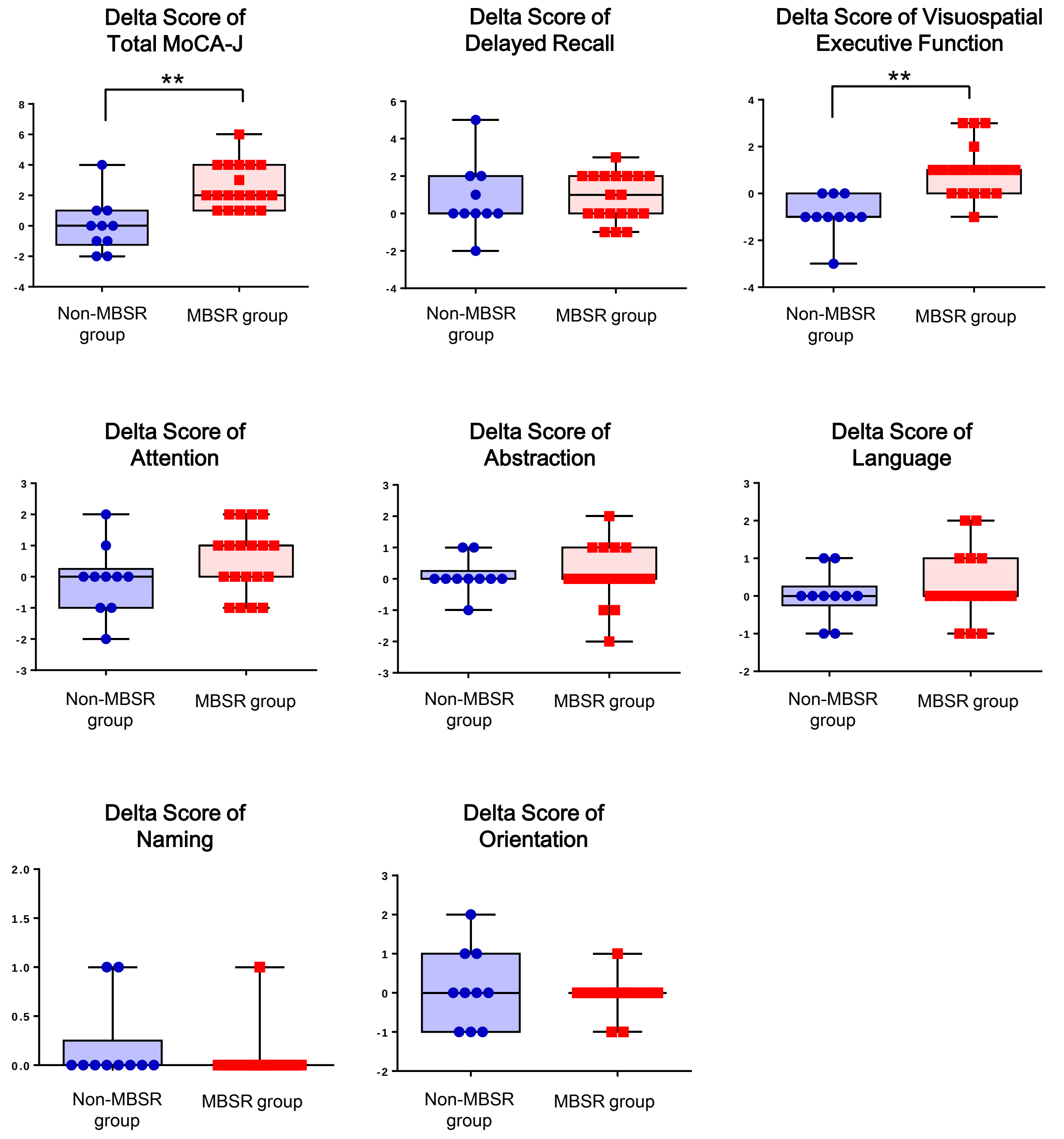

Supplementary Figure 1. The delta scores of the total MoCA-J and those scores of the seven domains (the score of post intervention – the score of pre intervention) were compared between the non-MBSR and MBSR group. The data were shown as box plot. $^{**}P < 0.01$, unpaired *t*-test. Non-MBSR group (n = 10), MBSR group (n = 19).

Supplementary Figure 2

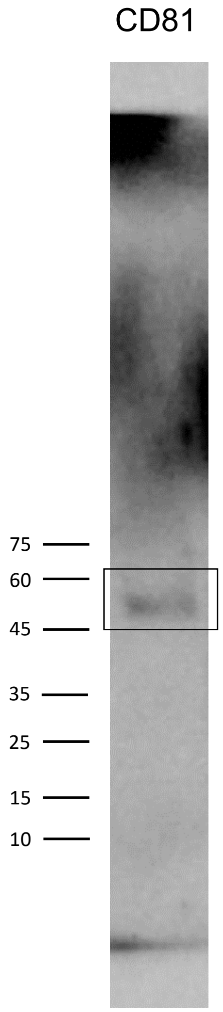

Supplementary Figure 2. The full-length blots in Figure 3.

Supplementary Figure 3

Position 1011-1017 of STAT3 3' UTR 5’ AGUUAACAGCCUCCUUGGUGCUU 3'
hsa-miR-29c-3p 3‘ AUUGGCUAAAGUUU- ACCACGAU 5’

Position 895-917 of STAT3 3' UTR (negative control: NC) 5’ AGGUUGCAGUGAGCCAAAAUUGC 3'
hsa-miR-29c-3p 3' AUUGGCUAAAGUUU- ACCACGAU 5’

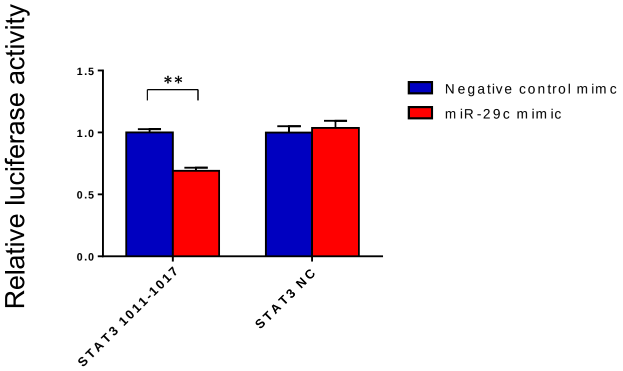

Supplementary Figure 3. The site on STAT3 targeted by miR-29c was predicted using TargetScan. Luciferase reporter assay verified that STAT3 is a target gene of miR-29c. Values are the means ± SD. The experiment was repeated four times. **P < 0.01, unpaired *t*-test. .

Supplementary Figure 4

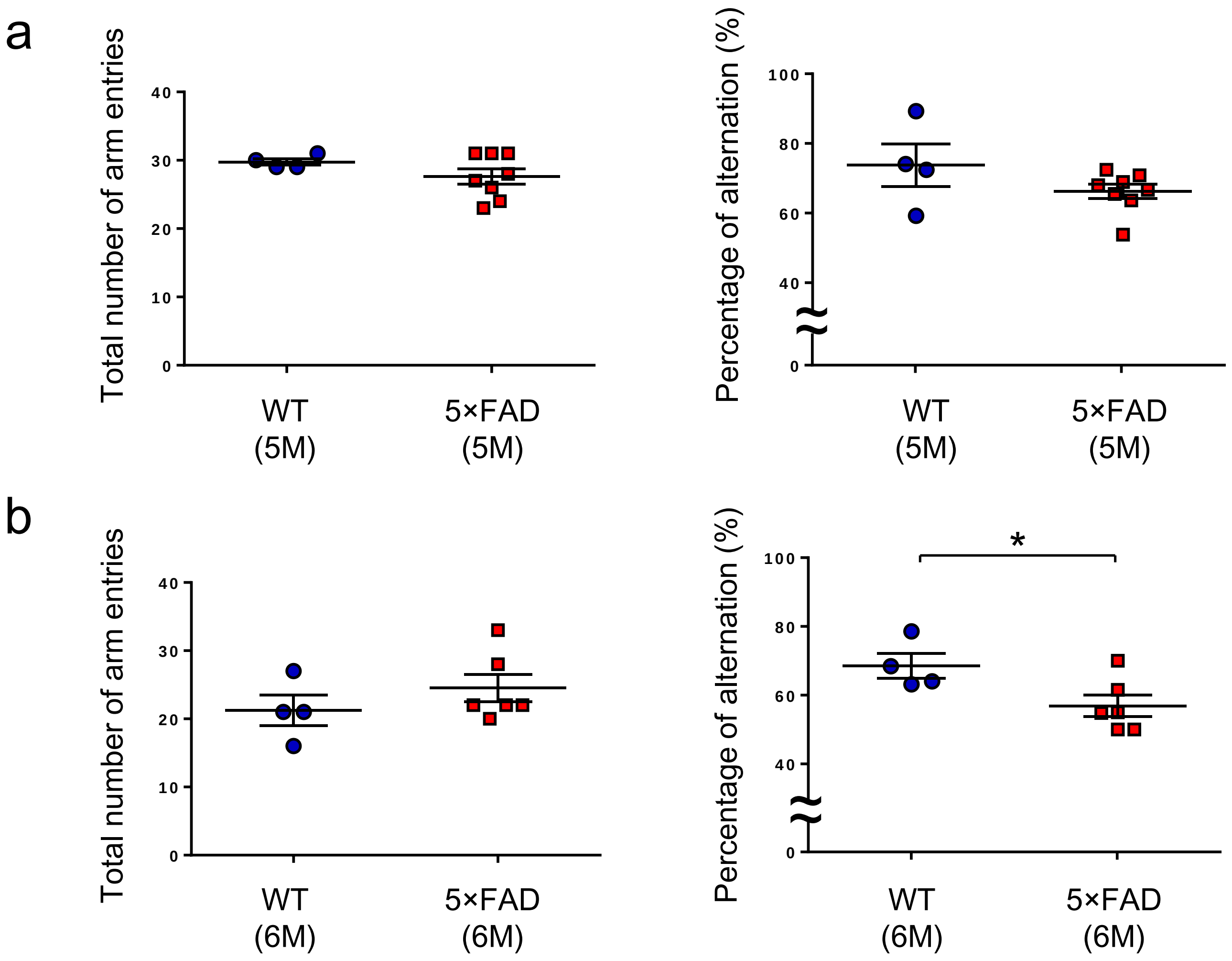

Supplementary Figure 4. (a) The results of the Y maze test of 5xFAD mice at 5 months old. Total numbers of arm entries and the percentages of alternations are shown. Values are the means ± SEM. Wild-type (WT) (n = 4), 5xFAD (n = 8). (b) The results of the Y maze test of 5xFAD mice at 6 months old. Total numbers of arm entries and the percentages of alternations are shown. Values are the means ± SEM. **P* < 0.05, unpaired *t*-test. WT (n = 4), 5xFAD (n = 6).
